# Effects of Maleic Anhydride-Grafted Polyethylene on the Properties of Artificial Marble Waste Powder/Linear Low-Density Polyethylene Composites with Ultra-High Filling Content

**DOI:** 10.3390/ma16114036

**Published:** 2023-05-29

**Authors:** Juncheng Die, Jianting Ma, Hai Li, Yafeng Zhang, Fei Li, Yang Cao, Wanjun Hao, Jinchun Tu, Kexi Zhang, Rentong Yu

**Affiliations:** State Key Laboratory of Marine Resource Utilization in South China Sea, School of Materials Science and Engineering, Hainan University, Haikou 570228, China; scho1ar@163.com (J.D.); majianting20011007@163.com (J.M.); lihai@hainanu.edu.cn (H.L.); zhangyafeng@hainanu.edu.cn (Y.Z.); 20085600210025@hainanu.edu.cn (F.L.); cy507@hainanu.edu.cn (Y.C.); hwj8899@163.com (W.H.); tujinchun@hainanu.edu.cn (J.T.)

**Keywords:** artificial marble waste powder, composites, maleic anhydride-grafted polyethylene, linear low-density polyethylene, mechanical properties

## Abstract

The need to reach carbon neutrality as soon as possible has made the use of recycled materials widespread. However, the treatment of artificial marble waste powder (AMWP) containing unsaturated polyester is a very challenging task. This task can be accomplished by converting AMWP into new plastic composites. Such conversion is a cost-effective and eco-friendly way to recycle industrial waste. However, the lack of mechanical strength in composites and the low filling content of AMWP have been major obstacles to its practical application in structural and technical buildings. In this study, a composite of AMWP/linear low-density polyethylene (LLDPE) filled with a 70 wt% AMWP content was fabricated using maleic anhydride-grafted polyethylene as a compatibilizer (MAPE). The mechanical strength of the prepared composites is excellent (tensile strength ~18.45 MPa, impact strength ~51.6 kJ/m^2^), making them appropriate as useful building materials. Additionally, laser particle size analysis, Fourier transform infrared spectroscopy, scanning electron microscopy, energy dispersive X-ray spectroscopy, and thermogravimetric analysis were used to examine the effects of maleic anhydride-grafted polyethylene on the mechanical properties of AMWP/LLDPE composites and its mechanism of action. Overall, this study offers a practical method for the low-cost recycling of industrial waste into high-performance composites.

## 1. Introduction

The announcement of the Chinese government’s carbon neutrality target is a milestone in the global response to climate change and a major commitment by China to construct sustainable communities, with far-reaching implications for global green and low-carbon development [1]. As the country pays more and more attention to ecological protection, natural marble mining is subject to many restrictions, and natural marble resources are increasingly scarce, leading to the production of many artificial marbles to replace the demand for natural marble [2]. As a substitute for natural marble, artificial marble has the characteristics of low cost, easy processing, and good performance and is widely used in architectural decoration, infrastructure construction, landscaping, and art materials [3,4,5,6,7,8,9]. Artificial marble was composed of CaCO_3_ and unsaturated polyester (mainly polyethylene terephthalate, PET) [10,11]. It will generate artificial marble waste powder (AMWP) during cutting, grinding, polishing, and other processing procedures. A large amount of discarded artificial marble is directly stacked and landfilled without treatment, which besides being a huge waste of resources, wastes a lot of land, pollutes groundwater, and affects people’s living environment, life safety, and property. Thus, recycling and utilization are necessary and imminent.

To date, some research has been conducted on the recycling and utilization of this waste. Hai Li et al. studied the replacement of cement fillers with different contents of AMWP. Their experimental results showed that since AMWP adsorbs on the surface of cement, the water reducer cannot be combined with cement, and free water cannot be released, resulting in a significant decrease in the fluidity of the cement slurry. On this basis, they introduced fatty acid methyl ester polyoxyethylene ether, which can be effectively adsorbed on the AMWP so that a water-reducing agent can enter into the cement, release free water, and improve the fluidity of the cement slurry [11].

Xin Huang et al. studied AMWP filled in polyvinyl alcohol (PVA). They showed that by adding AMWP, the intermolecular hydrogen bonds of PVA can be destroyed, and new hydrogen bonds are formed between PVA and the unsaturated polyester on the surface of AMWP, breaking old bonds and generation new bonds that can significantly increase the range of processing temperature of PVA, and improve the mechanical properties of composites [12].

Tej Singh et al. explored the co-filling of 15 wt% AMWP and different proportions of bagasse fibers into epoxy resin. The damping properties of the composites, such as storage modulus, loss modulus, and damping coefficient, were evaluated using a dynamic mechanical analyzer. Results showed that the composites with 10 wt% waste bagasse fiber loading exhibited the highest viscoelastic properties [9].

Polymer matrix composites are simple and inexpensive to manufacture and are considered to be the most fashionable commercial composites. One or several kinds of particles, which can improve performance and reduce costs, are used as fillers for composites. Linear low-density polyethylene (LLDPE) is a thermoplastic resin with good mechanical properties and chemical resistance [13,14]. It has been widely used in biology, agriculture, and industry [15,16,17,18].

Maleic anhydride grafted-polyethylene (MAPE) is an elastomer with excellent properties. It consists of several maleic anhydride molecules grafted on the polyethylene molecular chain [19,20,21]. It not only has the chemical inertness and processability of the saturated polyethylene molecular structure but also has the relatively strong chemical activity of the unsaturated maleic anhydride molecule; thus, it is a good compatibilizer.

Conventional pure inorganic substances such as CaCO_3_, kaolin, etc., are filled into the polymer, usually about 30%, to achieve the best mechanical properties [22,23,24,25,26]. When AMWP is blended with polymers, like pure inorganic substances, the hydrophilic AMWP is incompatible with the hydrophobic polymer matrix. The dispersibility of AMWP in the polymer is poor, the interfacial adhesion is weak, and the energy transfer from the polymer matrix to the stress-bearing AMWP particles is insufficient. As a result, the mechanical properties of the composites are reduced [27]. In addition, there is a lack of effective modification of the AMWP micro-interface, and the improvement of mechanical properties is insufficient.

In this study, the ultra-high filling of AMWP/LLDPE composites is demonstrated through molecular interface interaction and a plasticization strategy. Using MAPE as a compatibilizer, strong interfacial chemical bonds can be formed with the oxygen-containing groups of organics on the surface of AMWP. The anhydride groups of MAPE form hydrogen bonds with the polar groups at the AMWP interface, resulting in physical adsorption. Under the dual action of chemical bonding and physical adsorption, the interface compatibility between AMWP and the polyethylene surface is effectively improved, the dispersion of fillers is improved, and the mechanical properties of the composites are significantly improved. This approach provides new avenues for high-value waste reuse of AMWP for environmental and economic benefits.

## 2. Materials and Methods

### 2.1. Materials

Linear low-density polyethylene (LLDPE grade: DFDA-7042, density: 0.92 g/cm^3^, melt flow index: 2 g/10 min) was purchased from Ningxia Baofeng Energy Group Co., Ltd. (Yinchuan Ningxia, China). Artificial marble waste powder (AWMP) was purchased from Guangsha Building Materials Co., Ltd. (Hezhou, Guangxi, China). A Malvern MS2000 laser particle size analyzer (Malvern Ltd., Malvern, Worcestershire, UK) was used to analyze the particle size of AMWP. Maleic anhydride-grafted polyethylene (MAPE: density: 0.95 g/cm^3^, melt flow index is 2.3 g/10 min, and the grafting rate is 0.9 wt%) was purchased from Zhongjie Chemical Co., Ltd. (Guangzhou, Guangdong, China).

### 2.2. Preparation of AMWP/LLDPE Composites

To eliminate water, the AMWP was dried at 100 °C for 24 h. Using a JJ-5 high-speed mixer (Wuxi Jianyi Instrument & Machinery Co., Ltd., Wuxi, Jiangsu, China), LLDPE, AMWP, and MAPE were compounded in the ratios listed in Table 1.

The resulting mixture was then crushed into small pieces with a crusher and subsequently melt-mixed at 170 °C and 15 rpm for 30 min using an open mill for complete homogenization. Ultimately, the AMWP/LLDPE composites were molded into three-dimensional samples with an injection molding machine at the injection temperature of 180 °C, injection pressure of 10 MPa, mold temperature of 80 °C, and holding time of 15 s.

### 2.3. Characterization

#### 2.3.1. Sample Purification

The AMWP/LLDPE composites were immersed in analytical reagent (AR) grade xylene solution and stirred at 140 °C for 168 h, replacing the xylene solution every 12 h. Eventually, the residue was obtained by filtration and further purified using a Soxhlet extractor for 24 h to remove the polyethylene from the composites as well as the MAPE that was not chemically bonded [20].

#### 2.3.2. Fourier Transform Infrared (FTIR) Spectroscopy

Fourier transform infrared (FTIR) spectroscopy was performed on a Bruker Vertex 70 FTIR spectroscopy (Bruker Optik GmbH, Ettlingen, Germany) to record the FTIR spectra in the range from 4000 to 400 cm^−1^, with 16 scans, at a resolution of 2 cm^−1^. The samples were pressed into a thin film at 180 °C at 10 MPa pressure and then measured using the attenuated total reflection (ATR) mode.

#### 2.3.3. Scanning Electron Microscopy (SEM) and Energy Dispersive X-ray Spectroscopy (EDS)

The tensile cross-section morphology of microscopic AMWP and AMWP/LLDPE composites was examined by scanning electron microscopy (SEM) using a Verios G4, UC, Scanning Electron Microscope (Thermo Fisher Scientific Inc., Waltham, MA, USA). Briefly, after spraying the samples with gold, the surface morphology was observed under various magnifications while using a 20 kV accelerating voltage. Additionally, by turning the element Ca red, the Ca element distribution in the AMWP/LLDPE composites was ascertained. In this way, it was possible to determine the feature of AMWP dispersion in the AMWP/LLDPE composites.

#### 2.3.4. Thermogravimetric Analysis (TGA)

The thermal stability of the composites was determined by thermogravimetric analysis (TGA) to measure the thermal degradation of the AMWP/LLDPE composites, using a TGA Q600 Thermogravimetric Analyzer (TA Instruments Inc., New Castle, DE, USA). Briefly, approximately 8–10 mg of sample was heated from 30 to 60 °C at a ramp rate of 20 °C/min under a nitrogen atmosphere and held at the target temperature for 5 min to remove moisture. When the sample was cooled to 30 °C, it was heated to 900 °C with a ramp rate of 10 °C/min under a nitrogen atmosphere.

#### 2.3.5. Tensile Test

The tensile strength and elongation at break of the composites were determined according to the Chinese standard GB/T 1040-2006 for the determination of tensile properties of plastics, using AL-7000-SU2, universal testing machine (High-speed rail Instruments Inc., Taipei, Taiwan) with a crosshead speed of 50 mm/min and tensile specimen dimensions of 75 mm × 4 mm × 2 mm. The final test result is the median value of five sample measurements.

#### 2.3.6. Impact Test

The impact strength of cantilever beams without notches (80 mm × 10 mm × 4 mm) was determined according to the Chinese standard GB/T 1843-2008 for the determination of the impact strength of plastic cantilever beams using an XBJ-22 impact tester (Kaiqiang Instruments Inc., Shenzhen, Guangdong, China). The final test results were taken as the median value of five sample measurements.

## 3. Results and Discussion

### 3.1. Materials Design

**Figure 1:**
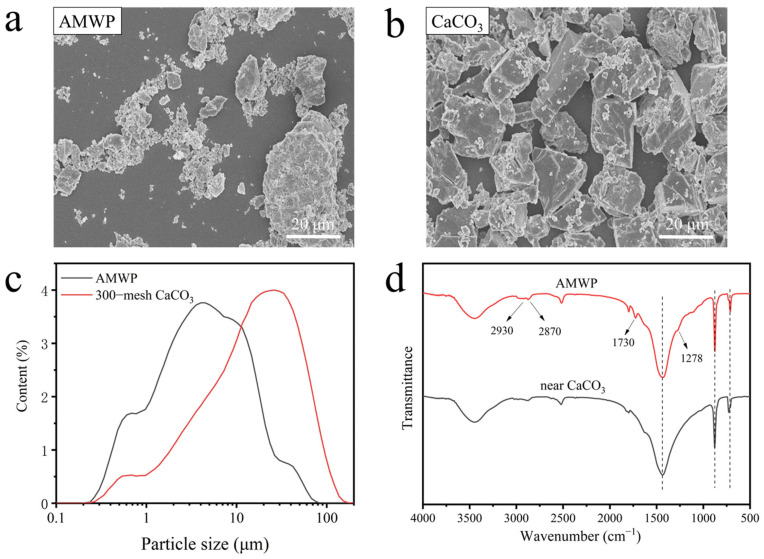
(**a**) SEM images of AMWP (**b**) SEM images of pure CaCO_3_. (**c**) Particle size distribution curve of AMWP and 300−mesh CaCO_3_. (**d**) FTIR spectra of AMWP and pure CaCO_3_.

Artificial marble is a non-degradable cross-linked polymer composite composed of CaCO_3_ and unsaturated polyester. The SEM images of AMWP and pure CaCO_3_, respectively, are shown in Figure 1a,b, respectively.

**Figure 2:**
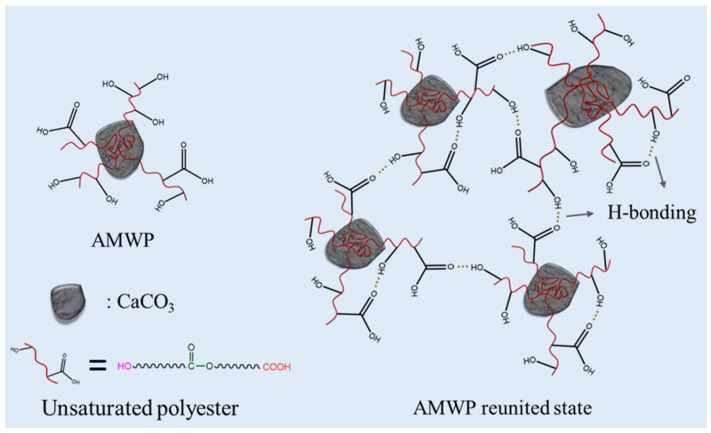
Schematic diagram of AMWP structure.

The surface of pure CaCO_3_ is flat and smooth, with well-defined edges and corners, and has a uniform particle size distribution. In contrast, AMWP particles are easily agglomerated, have a rough surface, are irregular and uneven in shape, and have an ideal specific surface area [19]. It was also found, from the particle size distribution curve in Figure 1c, that the median particle size of AMWP is 4.464 μm, but it is difficult to pass through the 300–mesh sieve. This may be caused by two reasons: thermodynamically speaking, AMWP particles are tiny, with large specific surface area and high specific surface area energy, while the energy tends to be in a lower direction. As a result, AMWP particles undergo an agglomeration phenomenon, making the surface energy lower. From chemical bonding, the unsaturated polyester on the surface of AMWP particles comes under the action of intra- and intermolecular hydrogen bonding, making AMWP particles agglomerate into one. In the FTIR spectra of AMWP and analytically pure CaCO_3_, shown in Figure 1d, the absorption bands at 2870 cm^−1^ and 2930 cm^−1^ in AMWP represent the symmetric and asymmetric stretching vibrations of the C–H bond [28], indicating that AMWP contains organic groups. The absorption bands at 1724 and 1278 cm^−1^ represent the C=O bond and C−O bond stretching vibrations, respectively, in the ester group [29], confirming the existence of the ester group in AMWP, thus indicating the presence of polyester. Since the hydroxyl stretching vibration of AMWP at 3445 cm^−1^ has a greater band intensity than that of pure CaCO_3_, this can be attributed to the terminal −OH and −COOH moieties in the polyesters [30]. These characteristic spectra also confirm that there are oxygen-containing groups such as −OH and −COOH on the surface of AMWP, which is expected to form interface interactions with the acid anhydride groups of MAPE. The AMWP structure is shown in Figure 2.

**Figure 3:**
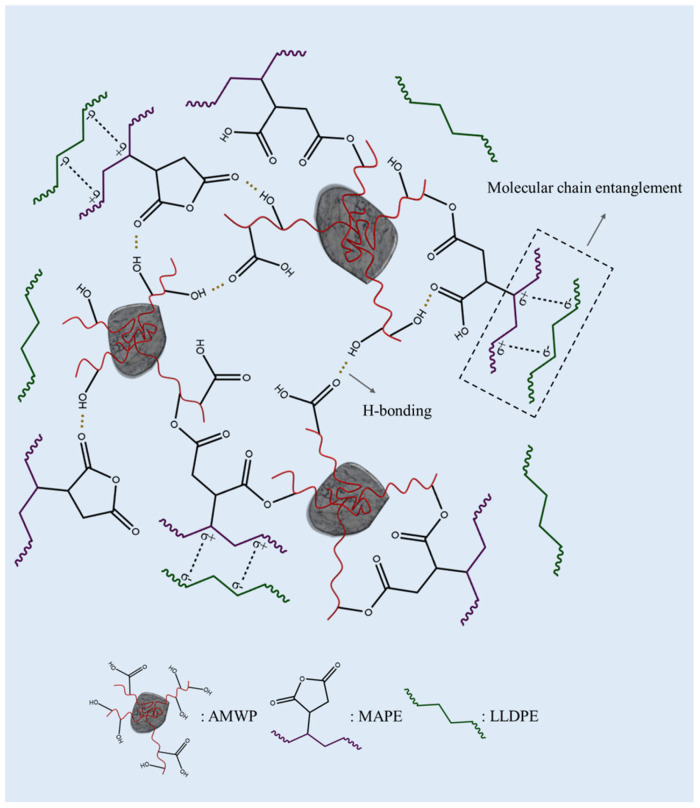
Mechanism of intermolecular complexation between MAPE and AMWP, and schematic diagram of the proposed supramolecular network with new hydrogen and ester bonds.

There are numerous hydroxyl and carboxyl groups in AMWP, which impart strong polarity to AMWP. Meanwhile, LLDPE is a non-polar thermoplastic polymer with certain crystallinity [31]. As a result, when AMWP and LLDPE are blended, there is incompatibility, the particles have weak adhesion at the interface, and the dispersion of AMWP in LLDPE is poor, resulting in lower filling content of AMWP and insufficient performance enhancement [32]. Based on chemical modification and the principle of “similar compatibility”, we designed and developed the AMWP/LLDPE composites with novel ester bonds and novel hydrogen bond networks, as shown in Figure 3, to achieve improved mechanical properties.

Briefly, on the one hand, the anhydride groups in MAPE are chemically cross-linked to form an ester bond with the hydroxyl group on the surface of AMWP [33,34,35], while the strong polarity of the anhydride group breaks the original hydrogen bonds of AMWP itself and creates new hydrogen bonds with the hydroxyl group in the unsaturated polyester on the surface of AMWP to form physical adsorption. On the other hand, the polyethylene chains in MAPE become entangled with the LLDPE chains during melt processing. Thus, the compatibility between AMWP and LLDPE is increased, and the mechanical properties of the composites can be enhanced.

### 3.2. FTIR Spectroscopy Analysis

In order to explore the cross-linking mechanism of action between MAPE and AMWP, xylene was used to remove the polyethylene remaining in the samples [20], and an FTIR spectroscopy analysis was performed on the treated sample.

**Figure 4:**
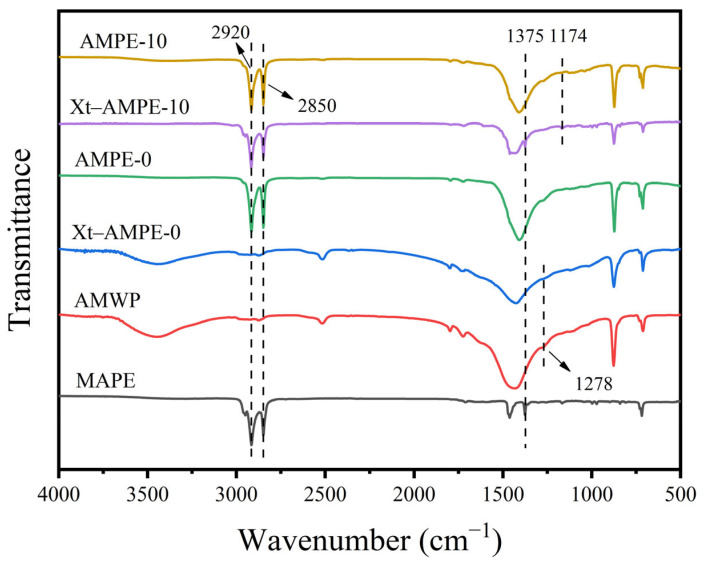
FTIR curves of MAPE, AMWP, and AMWP/LLDPE composites and their xylene-treated samples.

The FTIR spectra of pure MAPE, AMWP, AMPE-0, xylene-treated AMPE-0, AMPE-10, and xylene-treated AMPE-10 are shown in Figure 4.

The xylene-treated AMPE-0 showed the same absorption peak as AMWP, indicating that LLDPE was completely removed after the purification with xylene. However, the C=O absorption group at 1724 cm^−1^ and the stretching vibration group of the C-O bond in the ester group at 1278 cm^−1^ were weakened to varying degrees compared with AMWP, indicating that some of the polyesters were also dissolved in xylene. The xylene-treated AMPE-10 showed absorption peaks of −CH_2_ at 2920 cm^−1^ and 2850 cm^−1^ and −CH_3_ at 1375 cm^−1^, indicating that the molecular linkages of polyethylene branches of AMWP and MAPE were successful. Compared with AMPE-0, AMPE-10, and xylene-treated AMPE-10 showed a significant enhancement of the symmetric stretch peak at 1174 cm^−1^, indicating that the anhydride on MAPE breaks the bond and forms a new ester with the unsaturated groups on the surface of AMWP. Some studies have shown that the acid anhydride will undergo hydrolysis under the action of a catalyst during the melting process [34]. Specifically, with the incorporation of MAPE, AMWP may act as a catalyst to hydrolyze the acid anhydride groups on the MAPE molecular chain. In addition, the “free −OH and −COOH groups” of the unsaturated polyester molecules on the AMWP surface reacted with the hydrolyzed maleic anhydride chains, resulting in an esterification reaction. Thus, the AMWP is linked to the MAPE molecular chain.

### 3.3. Mechanical Properties of AMWP/LLDPE Composites

**Figure 5:**
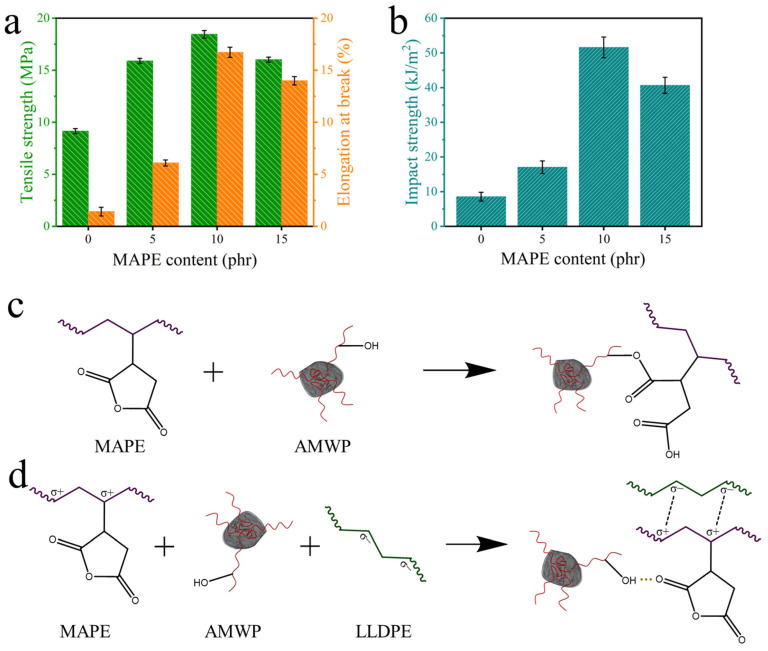
(**a**) Tensile strength and elongation at break of AMWP/LLDPE composites. (**b**) Impact strength of AMWP/LLDPE composites. (**c**) The chemical reaction between MAPE and AMWP. (**d**) Interaction forces between MAPE, AMWP, and LLDPE.

The MAPE-reinforced AMWP/LLDPE composites showed improved mechanical properties, as shown in Figure 5a,b, and Table 2.

The changes in tensile strength and elongation at the break of the AMWP/LLDPE composites with the increase of the amount of MAPE added are shown in Figure 5a. When the amount of MAPE added is 10 wt%, the tensile strength increases from 9.15 MPa to a maximum of 18.45 MPa, and the elongation at break also reaches a maximum of 16.92%, indicating that MAPE can impart good strength to the AMWP/LLDPE composites [36]. In contrast, the tensile strength of AMWP/LDPE composites made by conventional injection molding reached the highest value of 9.15 MPa at 10 wt% AMWP content [37].

The changes in the impact strength showed the same trend as tensile strength and elongation at break, as shown in Figure 5b. The impact strength of AMPE-0 is 8.57 kJ/m^2^ and reaches the highest value of 51.6 kJ/m^2^ when the amount of MAPE added is 10 wt%. The impact strength of AMWP/LDPE composites made by conventional injection molding reached the highest value of 28.98 kJ/m^2^ at 10 wt% AMWP content [37]. With the contribution of MAPE, the interfacial strength and toughness of the composites were optimized, which enhanced the resistance of AMWP/LLDPE composites to external forces and ultimately improved their impact strength. In addition, the molecular chain entanglement of MAPE and LLDPE inhibits the generation of silver streaks during the impact fracture process of the material and absorbs part of the energy during the fracture process, thereby optimizing the impact strength of AMWP/LLDPE composites [7,38,39]. The stress concentration due to the poor interaction (and cohesion) between MAPE and AMWP when the amount of MAPE added is more than 15 wt% is revealed by the sharp drop in the impact strength value, which decreased to 40.66 kJ/m^2^. Since energy absorption mainly occurs during deformation and fracture, these sharp drops in impact-absorbed energy are directly related to the extremely large drop in elongation at break. Therefore, this mechanical property improvement is related to not only the plasticization phenomenon but also various processes such as chain extension, compatibilization, branching, cross-linking, etc., due to the maleic anhydride groups which are highly reactive with the hydroxyl groups in AMWP.

The mechanical explanation of the performance enhancement of the AMWP/LLDPE composites by MAPE is illustrated in Figure 5c,d [40]. The interaction between MAPE and AMWP may occur due to two reasons. First, the acid anhydride groups on MAPE break the bond during the melting process with AMWP and undergo an esterification reaction with the hydroxyl groups in the unsaturated polyester on the surface of AMWP. Second, the strong polarity of the acid anhydride group destroys the intramolecular and intermolecular hydrogen bonds of AMWP and interacts with the hydroxyl groups on the surface of AMWP to form new hydrogen bonds and form intermolecular complexes. The interaction between MAPE and LLDPE occurs through the formation of entanglement or co-crystallization between the polyethylene backbone of MAPE and LLDPE. Under chemical and physical action, the mechanical properties of the composites were improved.

### 3.4. SEM and EDS Analysis of Tensile Sections of AMWP/LLDPE Composites

**Figure 6:**
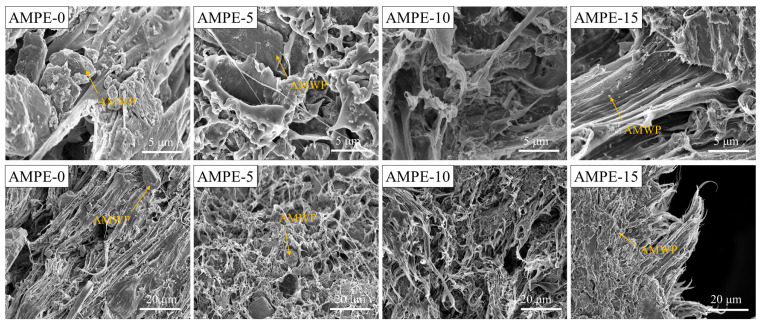
SEM images of tensile cross-sections of AMWP/LLDPE composites at different magnifications.

SEM images of the tensile section of the AMWP/LLDPE composites are shown in Figure 6.

When the amount of MAPE is added at 0 wt%, there are clear boundaries and gaps between AMWP and the polymer matrix, and AMWP is randomly distributed in LLDPE and remains intact without adhering to the matrix. This finding shows that, in the absence of MAPE, AMWP has poor interfacial adhesion on the surface of the polymer matrix, and the interfacial compatibility of the composites is poor. With the addition of MAPE, AMWP adheres to the surface of the polymer matrix and begins to cover the matrix, and the interface begins to blur. When the amount of MAPE added was 10 wt%, the AMWP almost completely covered the LLDPE matrix, and there were almost no free AMWP particles at the interface. This shows that the esterification reaction between the anhydride group of MAPE and the unsaturated polyester on the surface of AMWP is saturated, and the physical adsorption of MAPE and AMWP through hydrogen bonding has reached dynamic equilibrium. The branched chain of MAPE was grafted on the surface of AMWP to the maximum extent. When the amount of MAPE added was 15 wt%, AMWP was precipitated at the interface. This may be due to the excessive amount of MAPE, which makes the AMWP/LLDPE composite system polar and causes AMWP to agglomerate, thereby reducing the dispersion of AMWP in LLDPE.

**Figure 7:**
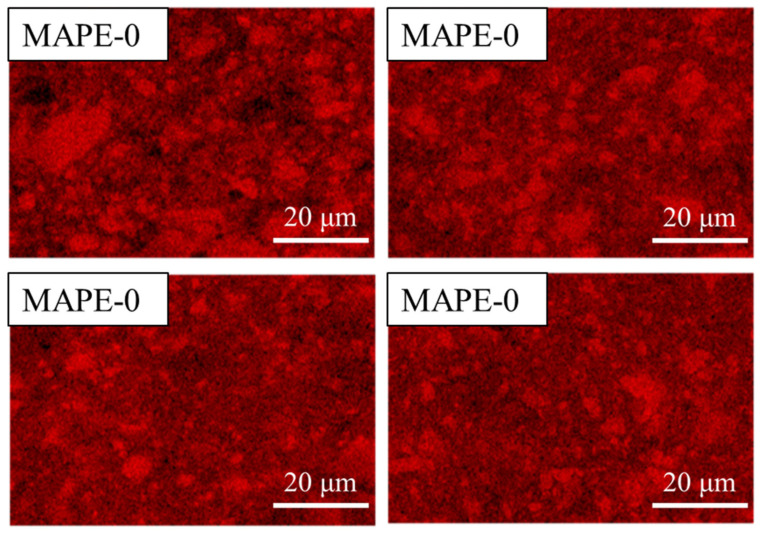
EDS diagram of a tensile section of AMWP/LLDPE composites.

In order to determine the dispersion of AMWP in LLDPE more intuitively [41], EDS elemental analysis was performed by setting the Ca element red, as shown in Figure 7.

The EDS analysis results show the same trend as the SEM results. There are many large dark-red, bright areas in the AMWP/LLDPE composites without MAPE, which indicates that the AMWP is aggregated and poorly dispersed in the LLDPE. With the addition of MAPE, the bright red area changes from large blocks to small blocks, and the color gradually becomes lighter. When the amount of MAPE added reaches 10 wt%, the block-like bright area is almost invisible, and AMWP is relatively uniformly dispersed without obvious agglomeration in the LLDPE matrix. When the amount of MAPE added exceeds 15 wt%, AMWP agglomerates again. The SEM images and EDS analysis results further demonstrated the excellent adhesion between AMWP and MAPE matrix and the strong interface interaction between the anhydride groups of MAPE and the hydroxyl groups on the AMWP surface.

### 3.5. Thermogravimetric Analysis

**Figure 8:**
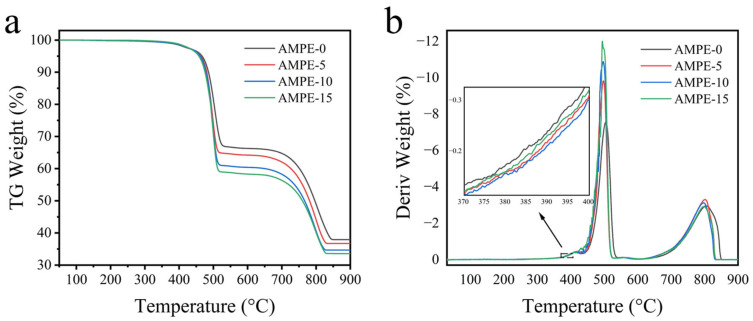
(**a**) AMWP/LLDPE composites TGA curve. (**b**) AMWP/LLDPE composites DTG curve.

According to the TGA curves of AMWP/LLDPE composites, shown in Figure 8a, the thermal degradation of AMWP/LLDPE composites occurs mainly in three stages.

The first decomposition peak in the range of 280–405 °C is attributed to the MAPE-polyester that has undergone chemical linkage [10]. The second decomposition peak in the range of 410–520 °C is assigned to the decomposition of the main chains of LLDPE and MAPE [42]. The third mass loss event, in the range of 630–825 °C, is attributed to the decomposition of CaCO_3_ [43]. Figure 8b is the derivative differential thermogravimetry (DTG) curve. With the addition of MAPE, the decomposition rate of the polyester on the AMWP surface is reduced due to the formation of ester bonds with AMWP. When the amount of MAPE added was higher than 15 wt%, the decomposition rate increased slightly. The improved thermal stability properties of MAPE also demonstrate the strong interface interaction between MAPE and AMWP.

## 4. Conclusions

MAPE can improve the mechanical properties of AMWP/LLDPE composites. Uncompatibilized composites have very poor polymer-particle interactions, resulting in poor tensile strength, elongation at break, and impact strength. With the addition of MAPE, the dispersion of AMWP in LLDPE is improved, and the tensile strength, elongation at break, and impact strength are enhanced. In AMWP/LLDPE composites, all these enhancements are attributed to the strong polarity and chemical instability of MAPE, as well as its effect on the uniform dispersion of AMWP in the LLDPE matrix and the impact of potential non-covalent interactions with other components. In this study, the mechanical properties were the most enhanced when the amount of MAPE added amount was 10 wt%. This study provides an effective approach for the resourceful and efficient utilization of artificial marble waste and an effective method for the preparation of high-performance composites for structural and engineering applications. This approach provides new avenues for high-value waste reuse of AMWP for environmental and economic benefits.

## Figures and Tables

**Table 1:** AMWP/LLDPE composites formulation.

Ingredients Phr	Formulation Code			
	AMPE-0	AMPE-5	AMPE-10	AMPE-15
AMWP	70	70	70	70
LLDPE	30	30	30	30
MAPE	0	5	10	15

**Table 2:** Mechanical properties of AMWP/LLDPE composites with different MAPE contents.

Sample	AMPE-0	AMPE-5	AMPE-10	AMPE-15
Tensile strength (MPa)	9.15 ± 0.24	15.89 ± 0.25	18.45 ± 0.36	16.01 ± 0.25
Elongation at break (%)	1.42 ± 0.41	6.09 ± 0.29	16.72 ± 0.49	13.99 ± 0.40
Impact strength (kJ/m^2^)	8.57 ± 1.26	17.06 ± 1.81	51.60 ± 2.99	40.66 ± 2.33

## Data Availability

Data openly available in a public repository.

## References

[1] Zhao Y., Su Q., Li B., Zhang Y., Wang X., Zhao H., Guo S. (2022). Have those countries declaring “zero carbon” or “carbon neutral” climate goals achieved carbon emissions-economic growth decoupling?. J. Clean. Prod..

[2] Uygunoğlu T., Topçu İ.B., Çelik A.G. (2014). Use of waste marble and recycled aggregates in self-compacting concrete for environmental sustainability. J. Clean. Prod..

[3] Ashish D.K. (2018). Feasibility of waste marble powder in concrete as partial substitution of cement and sand amalgam for sustainable growth. J. Build. Eng..

[4] Bilgin N., Yeprem H.A., Arslan S., Bilgin A., Günay E., Marşoglu M. (2012). Use of waste marble powder in brick industry. Constr. Build. Mater..

[5] Nayak S.K., Satapathy A., Mantry S. (2022). Use of waste marble and granite dust in structural applications: A review. J. Build. Eng..

[6] Ribeiro C.E.G., Rodriguez R.J.S., Vieira C.M.F., de Carvalho E.A., Candido V.S., Monteiro S.N. (2014). Production of synthetic ornamental marble as a marble waste added polyester composite. Mater. Sci. Forum.

[7] Chen H., He H., Tian S., Chen S. (2018). Recycling of waste artificial marble powder in HDPE-wood composites. Polym. Compos..

[8] Seghir N.T., Mellas M., Sadowski Ł., Żak A. (2018). Effects of marble powder on the properties of the air-cured blended cement paste. J. Clean. Prod..

[9] Singh T., Puri M., Tejyan S., Ravi R.K. (2021). Abrasive wear and dynamic–mechanical behavior of marble dust filled bagasse fiber reinforced hybrid polymer composites. Polym. Compos..

[10] Lu H., Chen K., Yang X., Liu J., Huang X., Lv Z., Zhang X. (2021). Use of titanate to improve interfacial interaction and mechanical properties of polyethylene/artificial marble wastes composites. J. Vinyl Addit. Technol..

[11] Li H., Ji D., Chen C., Wang X., Tu J., Zhang K., Ding A. (2021). Effect of fatty acid methyl ester polyoxyethylene ether on the rheological properties of cement filled with artificial marble waste powders. J. Clean. Prod..

[12] Huang X., Guo Q., Zhou P., Lu C., Yuan G., Chen Z., Zhang X. (2020). Poly (vinyl alcohol)/artificial marble wastes composites with improved melt processability and mechanical properties. Compos. Part B Eng..

[13] Jehanno C., Alty J.W., Roosen M., De Meester S., Dove A.P., Chen E.Y.-X., Leibfarth F.A., Sardon H. (2022). Critical advances and future opportunities in upcycling commodity polymers. Nature.

[14] Guo Q., Xue R., Zhao J., Zhang Y., van de Kerkhof G.T., Zhang K., Li Y., Vignolini S., Song D.P. (2022). Precise Tailoring of Polyester Bottlebrush Amphiphiles toward Eco-Friendly Photonic Pigments via Interfacial Self-Assembly. Angew. Chem. Int. Ed..

[15] Pearson A., Duncan M., Hammami A., Naguib H.E. (2022). Interfacial adhesion and thermal stability of high-density polyethylene glass fiber composites. Compos. Sci. Technol..

[16] Mészáros L., Tatár B., Toth K., Földes A., Nagy K.S., Jedlovszky-Hajdu A., Tóth T., Molnár K. (2022). Novel, injection molded all-polyethylene composites for potential biomedical implant applications. J. Mater. Res. Technol..

[17] Shi L., Hou Y., Chen Z., Bu Y., Zhang X., Shen Z., Chen Y. (2022). Impact of polyethylene on soil physicochemical properties and characteristics of sweet potato growth and polyethylene absorption. Chemosphere.

[18] Lu W., Yu W., Zhang B., Dou X., Han X., Cai H. (2021). Kevlar fibers reinforced straw wastes-polyethylene composites: Combining toughness, strength and self-extinguishing capabilities. Compos. Part B Eng..

[19] Wangtueai S., Chaiyaso T., Rachtanapun P., Jantrawut P., Ruksiriwanich W., Seesuriyachan P., Leksawasdi N., Phimolsiripol Y., Techapun C., Phongthai S. (2022). Thermoplastic cassava starch blend with polyethylene-grafted-maleic anhydride and gelatin core-shell structure compatibilizer. Int. J. Biol. Macromol..

[20] Hao X., Xu J., Zhou H., Tang W., Li W., Wang Q., Ou R. (2021). Interfacial adhesion mechanisms of ultra-highly filled wood fiber/polyethylene composites using maleic anhydride grafted polyethylene as a compatibilizer. Mater. Des..

[21] Jantanasakulwong K., Leksawasdi N., Seesuriyachan P., Wongsuriyasak S., Techapun C., Ougizawa T. (2016). Reactive blending of thermoplastic starch and polyethylene-graft-maleic anhydride with chitosan as compatibilizer. Carbohydr. Polym..

[22] Atli A., Noyel J.-P., Hajjar A., Antouly K., Lemaire E., Simon S. (2022). Exploring the mechanical performance of BaTiO_3_ filled HDPE nanocomposites: A comparative study of the experimental and numerical approaches. Polymer.

[23] Awad A., Abdel-Ghany A.W., Abd El-Wahab A.A., El-Gamasy R., Abdellatif M.H. (2020). The influence of adding marble and granite dust on the mechanical and physical properties of PP composites. J. Therm. Anal. Calorim..

[24] Awad A., Abdellatif M.H. (2019). Assessment of mechanical and physical properties of LDPE reinforced with marble dust. Compos. Part B Eng..

[25] Al-Samhan M., Al-Attar F. (2022). Comparative analysis of the mechanical, thermal and barrier properties of polypropylene incorporated with CaCO_3_ and nano CaCO_3_. Surf. Interfaces.

[26] Di X., Zhang Y., Zhang N., Ding C., Li Y., Zhang Y. (2022). Toughening action in marble tailings/PVC composite plates: Rheological and mechanical properties. Constr. Build. Mater..

[27] Ror C.K., Tejyan S., Kumar N. (2022). Effect of marble dust reinforcement in composites for different applications: A review. Mater. Today Proc..

[28] Liu Q., Xia C., He C., Guo W., Wu Z.P., Li Z., Zhao Q., Xia B.Y. (2022). Dual-Network Structured Hydrogel Electrolytes Engaged Solid-State Rechargeable Zn-Air/Iodide Hybrid Batteries. Angew. Chem..

[29] Mu X., Zhou J., Wang P., Chen H., Yang T., Chen S., Miao L., Mori T. (2022). A robust starch–polyacrylamide hydrogel with scavenging energy harvesting capacity for efficient solar thermoelectricity–freshwater cogeneration. Energy Environ. Sci..

[30] Mohit H., Mavinkere Rangappa S., Yorseng K., Siengchin S., Marwani H.M., Khan A., Asiri A.M. (2023). Discarded water hyacinth/pineapple fibers and carbon/innegra fabrics and TiC nanoparticles reinforced UV resistant polyester composites. J. Mater. Res. Technol..

[31] Cao B., Zhou Y., Wu Y., Cai J., Guan X., Liu S., Zhao J., Zhang M. (2020). Simultaneous improvement of processability and toughness of highly filled MH/LLDPE composites by using fluorine-containing flow modifiers. Compos. Part A Appl. Sci. Manuf..

[32] Azoug A., Nevière R., Pradeilles-Duval R.M., Constantinescu A. (2014). Influence of crosslinking and plasticizing on the viscoelasticity of highly filled elastomers. J. Appl. Polym. Sci..

[33] Song M.-X., Xie L.-J., Cheng J.-Y., Yi Z.-L., Song G., Jia X.-Y., Chen J.-P., Guo Q.-G., Chen C.-M. (2022). Insights into the thermochemical evolution of maleic anhydride-initiated esterified starch to construct hard carbon microspheres for Lithium-ion batteries. J. Energy Chem..

[34] Watanabe R., Sugahara A., Hagihara H., Mizukado J., Shinzawa H. (2020). Insight into interfacial compatibilization of glass-fiber-reinforced polypropylene (PP) using maleic-anhydride modified PP employing infrared spectroscopic imaging. Compos. Sci. Technol..

[35] Luo X., Wei Z., Seo B., Hu Q., Wang X., Romo J.A., Jain M., Cakmak M., Boudouris B.W., Zhao K. (2022). Circularly Recyclable Polymers Featuring Topochemically Weakened Carbon–Carbon Bonds. J. Am. Chem. Soc..

[36] Huang C.-W., Yang T.-C., Wu T.-L., Hung K.-C., Wu J.-H. (2018). Effects of maleated polypropylene content on the extended creep behavior of wood–polypropylene composites using the stepped isothermal method and the stepped isostress method. Wood Sci. Technol..

[37] Khan A., Patidar R., Pappu A. (2021). Marble waste characterization and reinforcement in low density polyethylene composites via injection moulding: Towards improved mechanical strength and thermal conductivity. Constr. Build. Mater..

[38] Huang C.-W., Yang T.-C., Hung K.-C., Xu J.-W., Wu J.-H. (2018). The effect of maleated polypropylene on the non-isothermal crystallization kinetics of wood fiber-reinforced polypropylene composites. Polymers.

[39] Gao H., Xie Y., Ou R., Wang Q. (2012). Grafting effects of polypropylene/polyethylene blends with maleic anhydride on the properties of the resulting wood–plastic composites. Compos. Part A Appl. Sci. Manuf..

[40] Li X., Yang Q., Zhang K., Pan L., Feng Y., Jia Y., Xu N. (2022). Property improvement and compatibilization mechanism of biodegradable polylactic acid/maleic anhydride-based/polypropylene spunbonded nonwoven slices. J. Clean. Prod..

[41] Song P., Xu Z., Lu Y., Guo Q. (2015). Bio-inspired hydrogen-bond cross-link strategy toward strong and tough polymeric materials. Macromolecules.

[42] Chaudhary A., Lakhani J., Dalsaniya P., Chaudhary P., Trada A., Shah N.K., Upadhyay D.S. (2023). Slow pyrolysis of low-density Poly-Ethylene (LDPE): A batch experiment and thermodynamic analysis. Energy.

[43] Zhu Y., Wang Z., Chen Z., Lin C., Li P., Huang Z., Cai P., Wu C., Zeng Q. (2022). High value-added utilization of artificial marble wastes and potential applications in biodegradable sustainable packaging. Compos. Sci. Technol..

